# Realization of the principles of the phenotypic prediction in the child caries prevention (Translation medicine experience in dental cariology)

**DOI:** 10.1186/1878-5085-5-S1-A114

**Published:** 2014-02-11

**Authors:** V Zagnat, V Okushko, V Ryabtsev

**Affiliations:** 1 TSU named after T.G. Shevchenko, Scientific Research Laboratory “STOMO” Tiraspol, Moldova

## 

It’s well known that dental caries is one of the common diseases worldwide. Dental caries is manifested by demineralization of tooth tissue and results from carbohydrate metabolism of microorganisms that reside in the oral cavity. While fluoride supplementation has helped reduce caries rate, implementation of modern principles of the translational medicine may also help to reduce the impact of dental caries. Studies carried out in twins suggest that about 80% of factors contributing to dental caries inheritable. Physiological factors and functional tooth resistance are linked with centrifugal transport of dental fluid exchange. The phenomenon of Dental Fluid Transport is suggested to be a primary mechanism of the resistance to dental caries.

The present study is intended to identify effective dental caries prohylaxis measures. Accordingly, the possibility of predictive personalized prevention of dental caries is studied on child population. In previous studies the bio-prediction test of dental enamel resistance (TER – test on enamel resistance) prior to dosed etching of enamel surface was used. It helped to identify the period of the year associated with decline of acid resistance of enamel (February - March) which is likely related to vitamin C. Subsequently, and based on these observations, a prophylaxis program was proposed and implemented. The program consisted of a 3 day administration of ascorbic acid in maximal doses for an age-specific group starting at the end of February. Yearly implementation of the program has reduced all the indicators of dental caries and has stabilized them at the point DMF 0.8 for the 12-year-old children. The program has included 100.000 subjects for 9 years. The results of the study have helped us to recommend a time-targeted predictive approach in dental caries prophylaxis programs.

Continued efforts should help with:

1. The establishment of unified diagnostic models for monitoring of persons from different dental caries risk groups by means of expanding of the bio-predictors including genotypic, allowing to make reasonable and appropriate diagnostics of preclinical process stages;

2. The development of pharmacy-prevention measures for correction of the decline in dental functional resistance, giving the ability to offer an individual protocol of the targeted personalized predictive treatment to a specific individual.

